# Supplementary material for: Large Area Fabrication of Semiconducting Phosphorene by Langmuir-Blodgett Assembly
Source: Sci Rep. 2016 Sep 27;6:34095. doi: 10.1038/srep34095 (PMC5037434; doi:10.1038/srep34095)
Supplement: Supplementary Information [file srep34095-s1.pdf]

Supplementary Information

for

Large Area Fabrication of Semiconducting Phosphorene by Langmuir-Blodgett Assembly

Harneet Kaur[1], Sandeep Yadav[2], Avanish. K. Srivastava[1], Nidhi Singh[1], Jörg J. Schneider[2], Om. P. Sinha[3], Ved V. Agrawal[1,+], Ritu Srivastava[1,+,*]

[1]National Physical Laboratory, Council of Scientific and Industrial Research, Dr. K. S. Krishnan Road, New Delhi 110012, India.
[2]Technische Universität Darmstadt, Eduard-Zintl-Institut für Anorganische und Physikalische Chemie L2 I 05 117, Alarich-Weiss-Str 12, 64287 Darmstadt, Germany.
[3]Amity Institute of Nanotechnology, Amity University, Sector 125, Noida, Uttar Pradesh 201313, India.

*Corresponding Author (Email: ritu@nplindia.org)
[+]Authors contributed equally in this work.

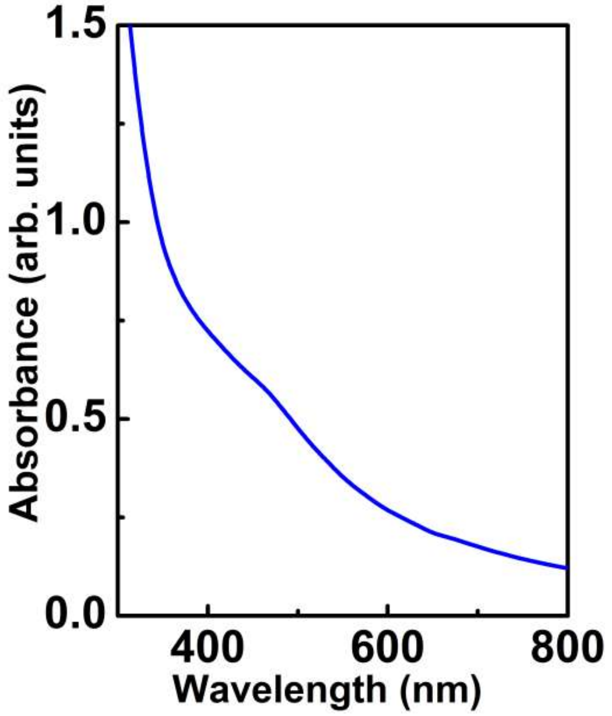

Figure S1: Optical absorbance spectra of exfoliated BP suspension in NMP, centrifuged at 3000 r.p.m.

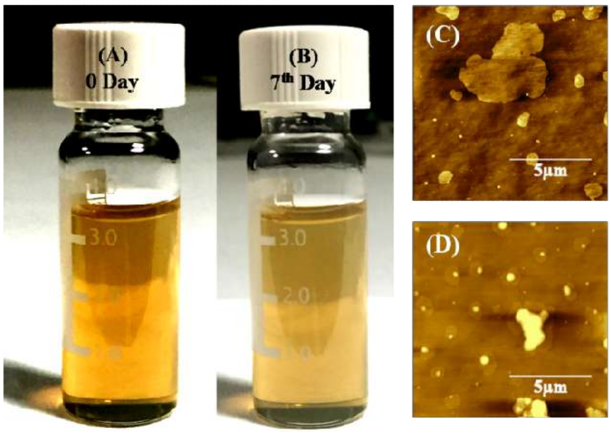

Figure S2: Digital image of phosphorene suspension and AFM of drop cast films. (A) Digital image on the first day of exfoliation. (B) Digital image after one week of ambient exposure showing the fading in the colour of suspension. (C) AFM of the drop-cast films prepared by using fresh exfoliated suspension. (D) AFM of the drop-cast films prepared by using one weak old suspension reveals the oxidation of nanosheets because of the formation of bubbles like structures on its surfaces.

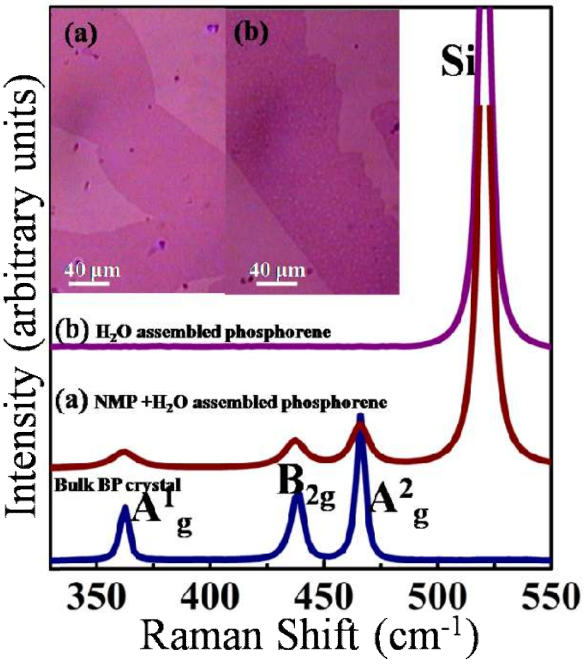

Figure S3: A comparison of the Raman spectra of bulk black phosphorus crystal (navy blue) and LB assembled phosphorene nanosheets (maroon and purple). The characteristic Raman modes are labelled. Inset (a): Confocal optical image of the nanosheet where Raman spectra was taken. These nanosheets were assembled using a mixture of NMP + deoxygenated water as subphase medium. The presence of Raman modes (maroon) and absence of bubbles in optical image suggests its pristine un-oxidized phase. Inset (b): Confocal

optical image of the nanosheet where Raman spectrum was taken. These nanosheets were assembled using deoxygenated water as a subphase medium. Presence of bubbles like structures on the surface of nanosheets in the optical image confirms its oxidation, resulting in absence of Raman vibrational modes (purple).

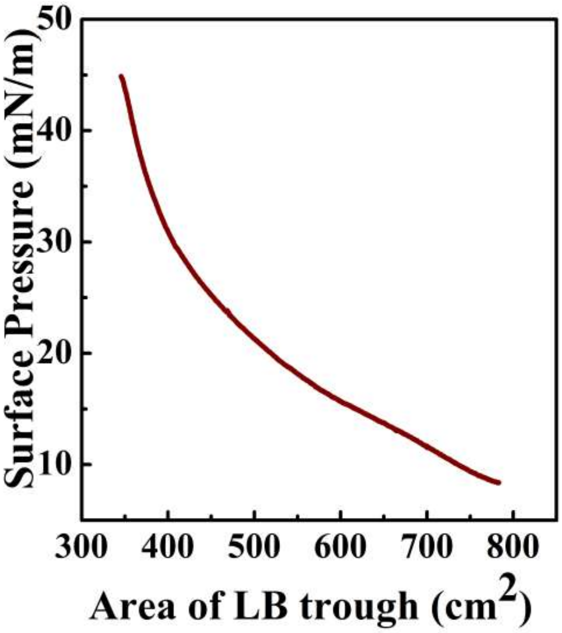

Figure S4: Variation in surface pressure with area of the LB trough on compressing the barriers. All the films were vertically lifted at a surface pressure of 40 mN/m.

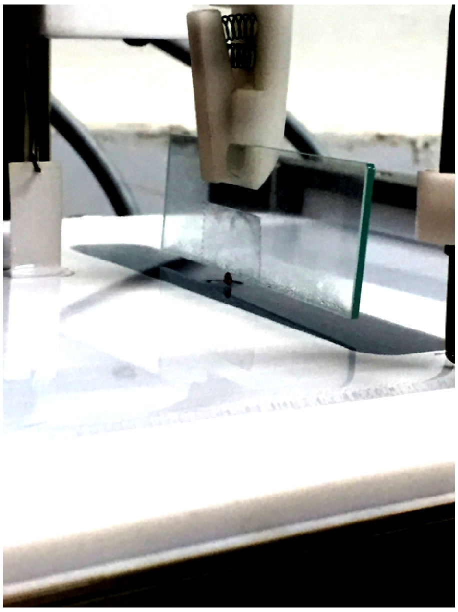

Figure S5: Digital image showing the vertical lift-off procedure on TEM grids in LB assembly.

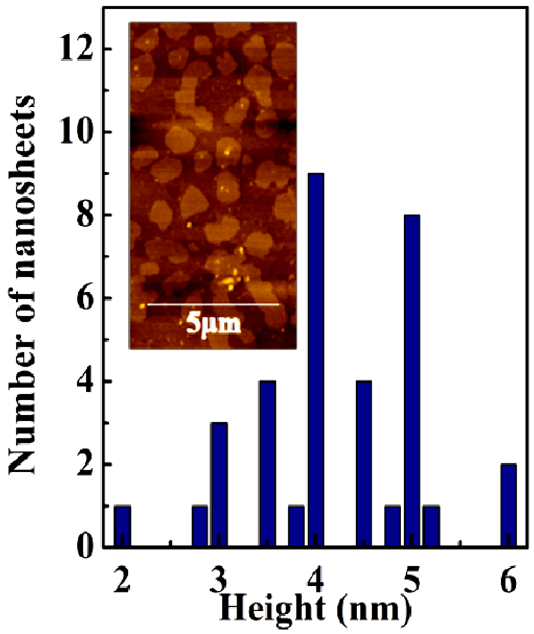

Figure S6: Height of the S-Ex BP nanosheets versus the number of sheets. Inset: AFM of the S-Ex BP nanosheets assembled by LB technique.

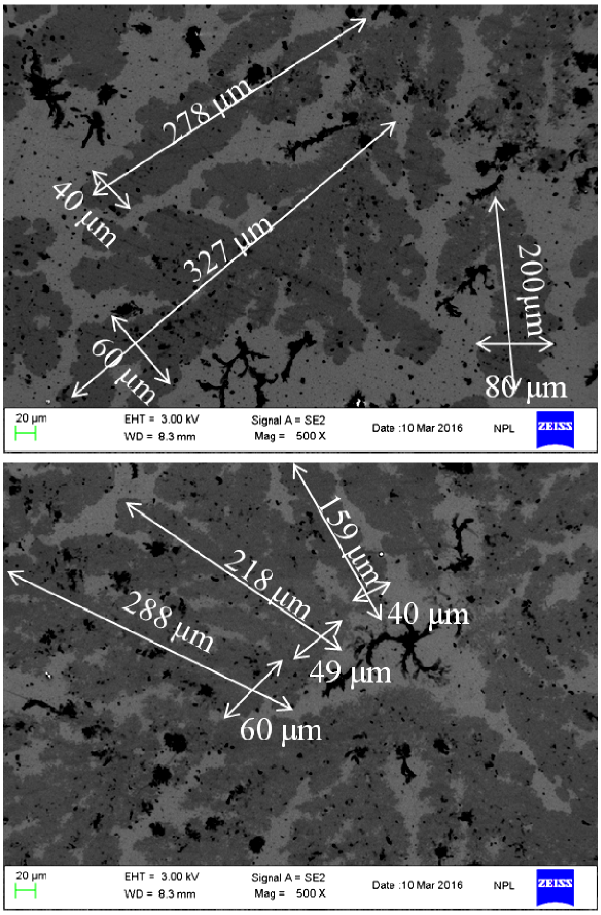

Figure S7: FESEM of LB assembled L-Ex BP nanosheets on SiO_2/Si substrate. The lateral dimension (defined as the largest side in our case) is of the order of hundreds of microns resulting in the enrichment of ultra-large nanosheets of phosphorene on substrate.
